# A TRIM21-based method for targeted protein degradation

**DOI:** 10.3724/abbs.2024179

**Published:** 2024-11-27

**Authors:** Weikang Hu, Xiang Qiu, Yun Yang, Yaoyao Wu, Chengcheng Wang, Ronggui Hu, Chuanyin Li

**Affiliations:** 1 Medical College of Guizhou University Guiyang 550025 China; 2 State Key Laboratory of Molecular Biology Shanghai Institute of Biochemistry and Cell Biology Center for Excellence in Molecular Cell Science Chinese Academy of Sciences Shanghai 200031 China; 3 Key Laboratory of Systems Health Science of Zhejiang Province School of Life Science Hangzhou Institute for Advanced Study University of Chinese Academy of Sciences Hangzhou 310024 China; 4 Department of Respiratory and Critical Care Medicine Guizhou Provincial People’s Hospital Guiyang 550002 China; 5 Department of Colorectal Surgery and Oncology (Key Laboratory of Cancer Prevention and Intervention China National Ministry of Education Key Laboratory of Molecular Biology in Medical Sciences Zhejiang Province China) the Second Affiliated Hospital Zhejiang University School of Medicine Hangzhou 310009 China

The ubiquitin-proteasome pathway is a highly selective protein degradation pathway that is capable of efficiently degrading intracellular proteins and plays an important role in various life processes [ 1, 2]. Dysfunction of this pathway has been associated with numerous problems, including cancer and neurodegenerative diseases [ 3, 4]. Targeted protein degradation (TPD) technologies have emerged as promising tools for use in a number of different areas, including biological research and clinical interventions [5]. Recently, a technology named Trim-Away was developed for the rapid degradation of proteins in mammalian cells [6]. Briefly, an antibody is designed against a target protein, and the E3 ligase TRIM21 is used to recognize the Fc region of the antibody and subsequently mediate antibody-dependent protein degradation via the proteasome ( Figure 1A).

To enhance the protein degradation efficiency of Trim-Away, three TRIM21-based constructs were designed: (1) deletion of the B-box domain of TRIM21, termed TRIM21 (ΔBB), (2) substitution of the RING domain of TRIM21 with the RING domain of MKRN1, termed TRIM21-RING. MKRN1 is a very potent E3 ligase, many of its substrates have been discovered to date, and it is also the E3 ligase we are currently using, and (3) substitution of the RING domain of TRIM21 with the HECT domain of UBE3A, designated TRIM21-HECT. UBE3A is the first E3 ligase with a HECT domain and is also the most extensively studied E3 ligase in our laboratory ( Figure 1B). The antibody was designed as a human IgG Fc region-fused nanobody ( Figure 1C), and the specific amino acid sequences of the nanobody used in this study can be found in the Supplementary Data. To test the protein degradation efficiency of these TRIM21-based constructs, plasmids encoding the d2EGFP, which is an EGFP destabilized by residues 422‒461 of mouse ornithine decarboxylase with an *in vivo* half-life of ~2 h, an antibody against d2EGFP (EGFP Fc-Nanobody), and various Trim21-based constructs were co-transfected into HEK293T cells. EGFP expression was determined by immunoblotting and fluorescence microscopy. The results revealed that TRIM21 (ΔBB) exhibited the most effective degradation performance, followed by TRIM21, whereas TRIM21-RING and TRIM21-HECT performed poorly ( Figure 1D,E). A dose-dependent assay of these TRIM21-based constructs was then performed, and again, TRIM21 (ΔBB) showed the best degradation performance, even at lower doses, compared with the other three constructs ( Figure 1F).

Human papillomavirus (HPV), with more than 200 known HPV subtypes, is a major contributor to the global burden of cancer. High-risk HPV subtypes, including HPV16 and HPV18, are associated with the development of approximately 90% of cervical cancers [7]. Two viral oncoproteins, HPV early protein 6 (E6) and early protein 7 (E7), have been shown to play a role in carcinogenesis [7]. The removal of E6 or E7 represents a potential method for the treatment of associated tumors. Antibodies against E6 and E7 (E6/E7 Fc-Nanobody) were designed and initially validated for their ability to degrade Trim21-based TPD constructs on the HPV E6 and E7 proteins in HEK293T cells with exogenously expressed E6 and E7. The results showed that TRIM21 (ΔBB) exhibited the most effective degradation effect ( Figure 2A,B). The effects of these constructs on the E6 and E7 proteins were then investigated in the human cervical cancer cell line CaSki (HPV16 positive). All the constructs effectively mediated the degradation of the E6 and E7 proteins, with TRIM21 (ΔBB) showing the most pronounced effect ( Figure 2C,D). Further investigation revealed that the TRIM21 (ΔBB) construct was able to degrade the E6 and E7 proteins in a dose-dependent manner ( Figure 2E,F). Therefore, the TRIM21 (ΔBB) system was selected for further study.

E6AP/UBE3A, the founding member of the HECT-type ubiquitin ligase family, was first identified as the viral E6 partner of HPV [8]. HPV 16 E6 hijacks the ligase activity of the host E6AP protein to ubiquitinate and subsequently degrade the p53 tumor suppressor protein [9]. The TRIM21 (ΔBB) system mediated the degradation of E6, which activated the expression of p53 and its downstream molecule p21 ( Figure 2G), which was consistent with the finding that p21 luciferase activity was significantly promoted by the TRIM21 (ΔBB) system ( Figure 2H). Further studies revealed that the TRIM21 (ΔBB) system inhibited the proliferation of human cervical cancer CaSki cells, as determined by the Cell Counting Kit-8 assay and the colony formation assay ( Figure 2I,J).

Taken together, our findings indicated that the TRIM21 (ΔBB) system is a more effective method of targeted protein degradation than the wild-type TRIM21-based system, which is able to mediate the degradation of EGFP, E6 and E7 more efficiently than the wild-type TRIM21-based system ( Figure 2K). Notably, other studies have also shown that deletion of the B-box domain of TRIM21 can improve its efficiency for targeted protein degradation [10]. One study has shown that TRIM21 is auto-regulated via the B-box domain, as deletion of the B-box increases the catalytic activity of its RING domain [11]. This may explain why deletion of the B-box enhances the capacity of TRIM21 for TPD.

How can the antibody be efficiently delivered to the cell? A number of technologies have been developed. These strategies can be grouped into several broad classes: direct physical delivery, direct intracellular expression, fusion with the internal autoantibody responsible for their intrinsic ability to enter cells, the use of protein transduction domains or their mimics, and the use of various nanoparticle carriers [12]. In this study, we chose the simplest strategy, direct intracellular expression.

We are also trying to develop other TRIM21-based systems for targeted protein degradation, such as TRIM21-RING and TRIM21-HECT, but unfortunately, they do not work well in the d2EGFP test, even less so than wild-type TRIM21. Perhaps because they are engineered fusion proteins, there are some problems with their advanced structure that affect their catalytic activity. Surprisingly, TRIM21-HECT is able to degrade E6 and E7 efficiently, which needs to be investigated further. Although only E6 and E7 were tested in this study, we believe that the TRIM21 (ΔBB) system may be suitable for more scenarios.

**Figure 1:**
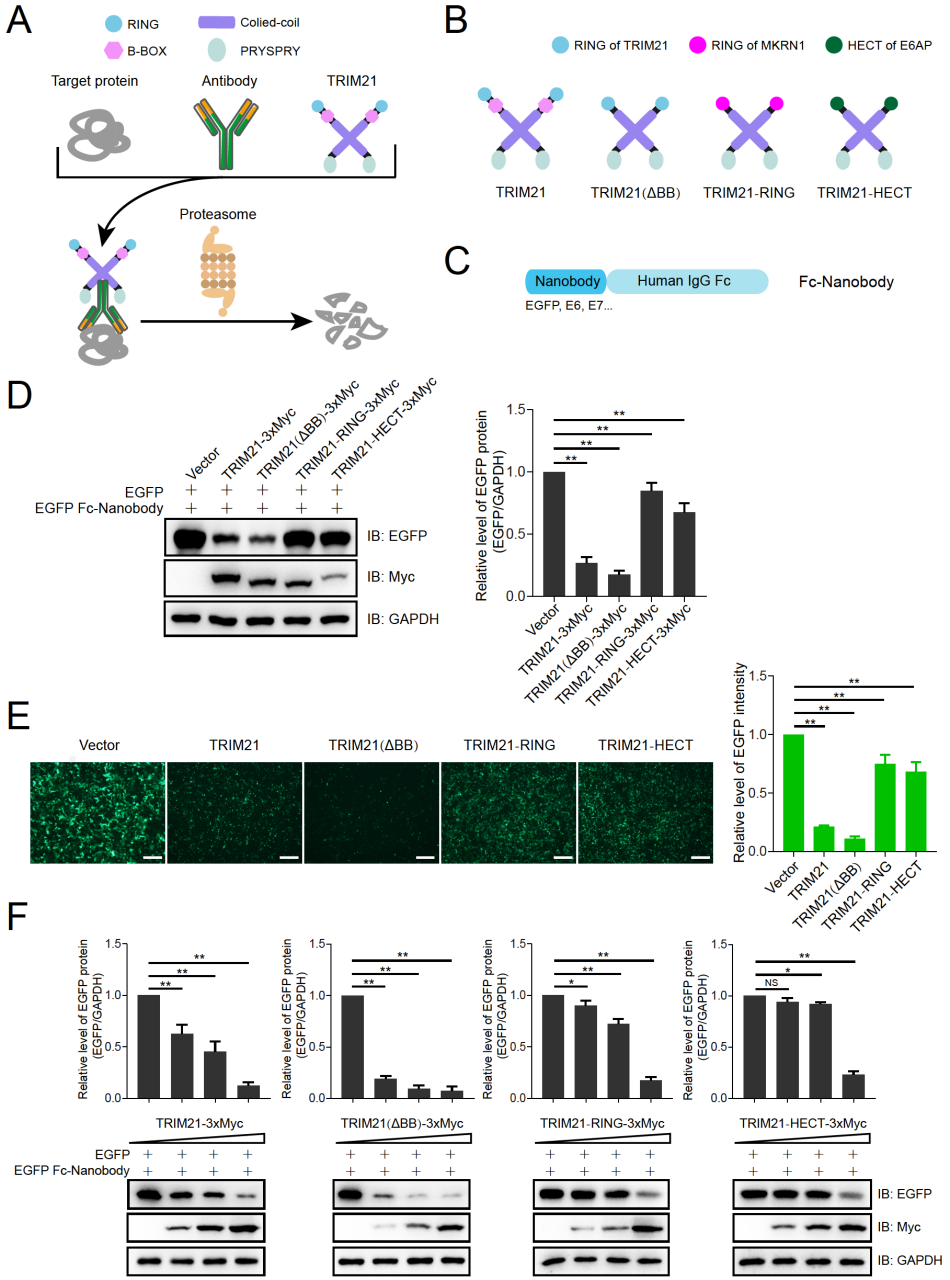
Figure 1 Engineering the E3 ubiquitin ligase TRIM21-based targeted protein degradation system and validation (A) Schematic of the wild-type TRIM21-based targeted protein degradation (TPD) system. (B) Design of TRIM21-based TPD constructs. (C) The antibody used for the TRIM21-based TPD system, named Fc-Nanobody. (D,E) Validation of the degradation efficiency of TRIM21-based TPD constructs for the d2EGFP. HEK293T cells were co-transfected with plasmids encoding d2EGFP, EGFP Fc-Nanobody and different Myc-tagged TRIM21-based TPD constructs. EGFP expression was determined by immunoblotting (D) or fluorescence microscopy (E) 48 h after transfection and quantified using Image J. This experiment was repeated three times. Scale bar: 300 μm; **P < 0.01. (F) Detection of the degradation efficiency of TRIM21-based TPD constructs in a dose-dependent manner. HEK293T cells were co-transfected with plasmids encoding d2EGFP or the EGFP Fc nanobody and different amounts (0, 1, 2, or 4 μg) of Myc-tagged TRIM21-based TPD constructs. EGFP expression was determined by immunoblotting 48 h after transfection and quantified using Image J. This experiment was repeated three times. NS, no significant difference; *P < 0.05, **P < 0.01.

**Figure 2:**
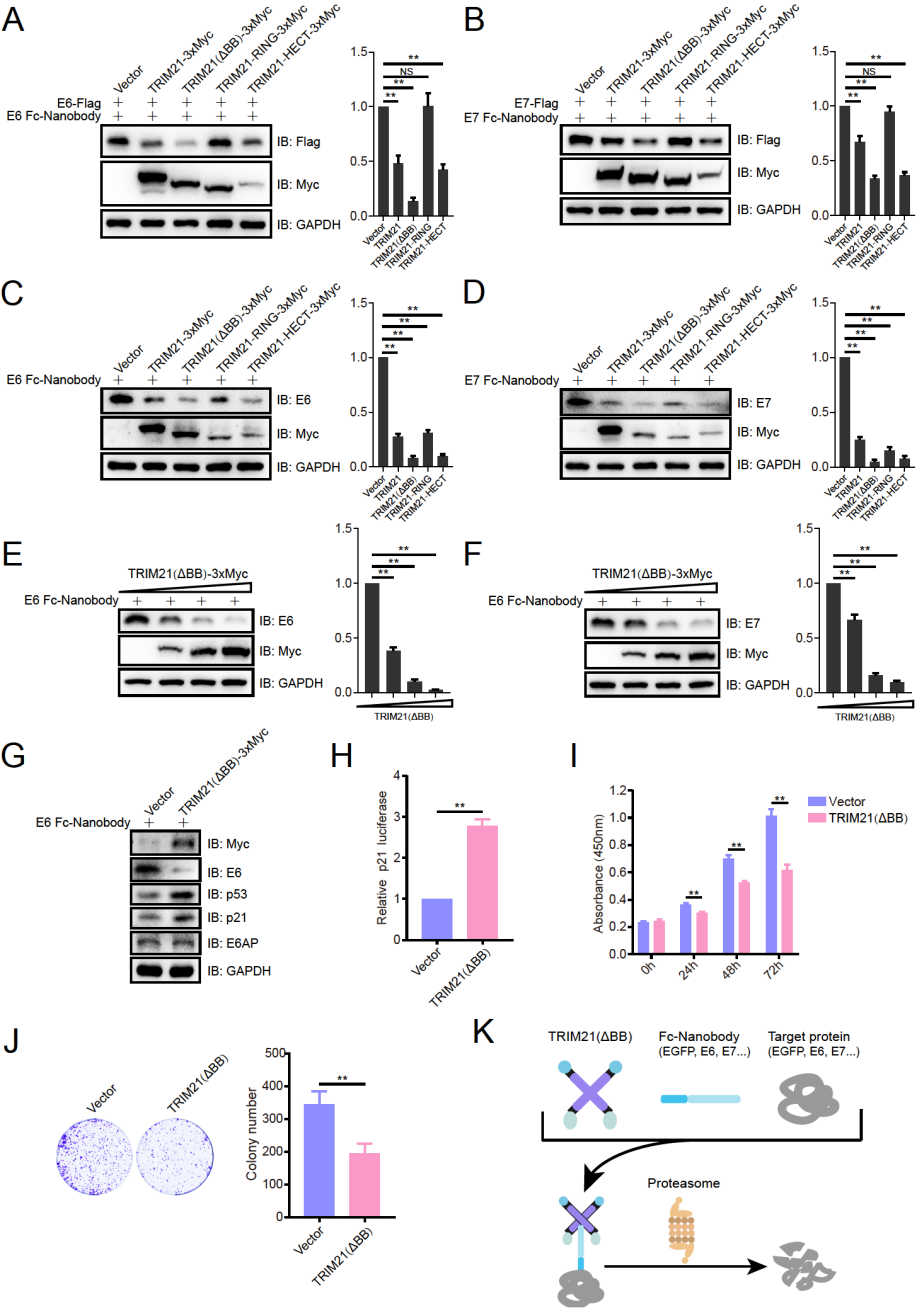
Figure 2 The TRIM21 (ΔBB) system is efficient for human papillomavirus 16 (HPV-16) E6 protein degradation and inhibits the proliferation of human cervical cancer cells (A,B) Validation of the degradation efficiency of TRIM21-based TPD constructs on exogenous HPV E6 and E7 proteins. HEK293T cells were co-transfected with plasmids encoding Flag-tagged E6/E7, E6/E7 Fc-Nanobody and different Myc-tagged Trim21-based TPD constructs. E6 or E7 expression was determined by immunoblotting with an anti-Flag antibody 48 h after transfection. NS, no significant difference; **P < 0.01. (C,D) Determination of the degradation efficiency of TRIM21-based TPD constructs on the HPV E6 and E7 proteins. Human cervical cancer CaSki cells were co-transfected with plasmids encoding E6/E7 Fc-Nanobodies and different Myc-tagged Trim21-based TPD constructs. E6 or E7 expression was determined by immunoblotting 48 h after transfection. **P < 0.01. (E,F) The TRIM21 (ΔBB) system degraded the E6 and E7 proteins in a dose-dependent manner. CaSki cells were co-transfected with plasmids encoding E6/E7 Fc-Nanobody and different amounts (0, 1, 2, or 4 μg) of Myc-tagged TRIM21 (ΔBB) constructs. E6 or E7 expression was determined by immunoblotting 48 h after transfection. **P < 0.01. (G) E6 degradation by the TRIM21 (ΔBB) system activated the p53-p21 signaling pathway. CaSki cells were co-transfected with plasmids encoding E6 Fc-Nanobody and Myc-tagged TRIM21 (ΔBB), and the levels of E6, p53, p21 and other indicated proteins were detected by immunoblotting 48 h after transfection. (H) p21 luciferase activity was promoted by the TRIM21 (ΔBB) system. CaSki cells were co-transfected with pGL3-p21-luc, pRL-TK and plasmids encoding E6 Fc-Nanobody and TRIM21 (ΔBB) for 48 h before being subjected to a luciferase activity assay. This experiment was repeated three times in triplicate. **P < 0.01. (I) The TRIM21 (ΔBB) system inhibits the proliferation of human cervical cancer cells. The viability of CaSki cells co-transfected with plasmids encoding the E6 Fc-Nanobody and TRIM21 (ΔBB) was assessed using a Cell Counting Kit-8 assay at different time points (0, 24, 48, and 72 h). The 0 h time point was defined as 6 h after cell seeding. This experiment was repeated three times with six replicates. **P < 0.01. (J) The TRIM21 (ΔBB) system inhibits the colony formation of human cervical cancer cells. CaSki cells were co-transfected with plasmids encoding E6 Fc-Nanobody and TRIM21 (ΔBB) and then seeded in 6-well plates. A colony formation assay was then performed. The number of colonies was counted and calculated. **P < 0.01. (K) Schematic of the TRIM21 (ΔBB) system for protein degradation.

## Supplementary Data

Supplementary data is available at *Acta Biochimica et Biophysica Sinica* online.

Research_Highlights
