# Supplementary material for: A TRIM21-based method for targeted protein degradation: TRIM21-based method for targeted protein degradation
Source: Acta Biochim Biophys Sin (Shanghai). 2024 Nov 27;57(4):667–70. doi: 10.3724/abbs.2024179 (PMC12040746; doi:10.3724/abbs.2024179)
Supplement: Research_Highlights [file 24616Supplementary_materials-z.docx]

**Supplementary Materials and Methods**

**Plasmid construction**

The full-length wild-type TRIM21 was amplified using human HEK29T cDNA and inserted into the pCDNA3.0 vector with a 3′ 3×Myc tag using the Seamless Cloning kit (Beyotime Biotechnology, Shanghai, China). TRIM21 (ΔBB) was constructed using wild-type TRIM21 as a template by site-directed mutagenesis as previously described [1]. The TRIM21-RING construct was generated by substituting the RIING domain of TRIM21 with the RIING domain of MKRN1 using the Seamless Cloning kit. The TRIM21-HECT construct was generated by replacing the RIING domain of TRIM21 by the HECT domain of UBE3A using the Seamless Cloning kit.

The antibody was designed as a human IgG Fc region-fused nanobody, which was subsequently inserted into the pCDNA3.0 vector. The amino acid sequences of the human IgG Fc and nanobodies are listed below:

(1) Human IgG Fc: DKTHTCPPCPAPELLGGPSVFLFPPKPKDTLMISRTPEVTCVVVDVSHEDPEVKFNWYVDGVEVHNAKTKPREEQYNSTYRVVSVLTVLHQDWLNGKEYKCKVSNKALPAPIEKTISKAKGQPREPQVYTLPPSREEMTKNQVSLTCLVKGFYPSDIAVEWESNGQPENNYKTTPPVLDSDGSFFLYSKLTVDKSRWQQGNVFSCSVMHEALHNHYTQKSLSLSPG;

(2) EGFP nanobody: MADVQLVESGGGLVQAGGSLRLSCAASGRTISMAAMSWFRQAPGKEREFVAGISRSAGSAVHADSVKGRFTISRDNTKNTLYLQMNSLKAEDTAVYYCAVRTSGFFGSIPRTGTAFDYWGQGTQVTVS;

(3) E6 nanobody: MDVQLVESGGGLVQPGGSLRLSCAASGFTFGDYGMSWVRQAPGKGLEWVSNIRSGPDSTNYADSVKGRFTISRDDSKSTLYLQMNSLKPEDTATYYCATDRGGRTRRGQGTQVTVSS;

(4) E7 nanobody: QVQLVESGGGSVQAGGSLRLSCAASGYTSSSCSMGWYRQAPGKERELVATIFADGRTRYADSVKGRFTISRDNAKNTVYLQMNSLKPEDTAMYYCNTDAHGSYSDYDCVNWNNYWGQGTQVTVSS.

The plasmid containing d2EGFP was kindly provided by Prof. Haifeng Ye (East China Normal University, Shanghai, China). Human papillomavirus (HPV) E6 and E7 cDNAs were synthesized and inserted into the pcDNA3.0 vector with a 3′ flag tag. The amino acid sequences of E6 and E7 are listed below:

(1) d2EGFP:

MVSKGEELFTGVVPILVELDGDVNGHKFSVSGEGEGDATYGKLTLKFICTTGKLPVPWPTLVTTLTYGVQCFSRYPDHMKQHDFFKSAMPEGYVQERTIFFKDDGNYKTRAEVKFEGDTLVNRIELKGIDFKEDGNILGHKLEYNYNSHNVYIMADKQKNGIKVNFKIRHNIEDGSVQLADHYQQNTPIGDGPVLLPDNHYLSTQSALSKDPNEKRDHMVLLEFVTAAGITLGMDELYKKLSHGFPPEVEEQDDGTLPMSCAQESGMDRHPAACASARINV;

(2) E6: MHQKRTAMFQDPQERPRKLPQLCTELQTTIHDIILECVYCKQQLLRREVYDFAFRDLCIVYRDGNPYAVCDKCLKFYSKISEYRHYCYSLYGTTLEQQYNKPLCDLLIRCINCQKPLCPEEKQRHLDKKQRFHNIRGRWTGRCMSCCRSSRTRRETQL;

(3) E7: MHQKRTAMFQDPQERPRKLPQLCTELQTTIHDIILECVYCKQQLLRREVYDFAFRDLCIVYRDGNPYAVCDKCLKFYSKISEYRHYCYSLYGTTLEQQYNKPLCDLLIRCINCQKPLCPEEKQRHLDKKQRFHNIRGRWTGRCMSCCRSSRTRRETQL.

**Cell culture and transfection**

The human embryonic kidney cell line 293T (HEK293T) and the cervical cancer cell line CaSki (HPV16-positive) were obtained from the Cell Bank of the Chinese Academy of Science (Shanghai, China), cultured in high-glucose DMEM supplemented with 10% fetal bovine serum (FBS; Gibco, Carlsbad, USA), 100 mg/mL streptomycin and 100 U/mL penicillin (Gibco), and placed in a humidified incubator with 5% CO_2_ at 37°C. The plasmids were transfected into the cells using Lipofectamine 2000 (Life Technologies, Carlsbad, USA) according to the manufacturer’s instructions.

**Immunoblotting (IB)**

Cells transfected with the indicated plasmids were lysed in RIPA buffer [50 mM Tris-HCl, 150 mM NaCl, 5 mM EDTA, 0.1% SDS (sodium dodecyl sulfate), and 1% NP-40, pH 7.5] supplemented with a protease inhibitor cocktail (1:100; Selleck, Houston, USA). The supernatant was then collected by centrifugation at 12,000 *g* for 10 min at 4°C and denatured at 100°C for 20 min in 1× SDS-PAGE protein loading buffer. Denatured cell lysates were subjected to SDS-PAGE and transferred to 0.22-µm PVDF membranes (Millipore, Billerica, USA). The membranes were blocked with 10% nonfat milk for 30 min at room temperature before incubation with the following antibodies: anti-EGFP (1:1000, 31002ES20; Yeasen, Shanghai, China), anti-GAPDH (1:10,000, 60004-1-Ig; Proteintech, Wuhan, China), anti-Myc (1:8000, 16286-1-AP; Proteintech), anti-E6 (1:500, ab20192; Abcam, Cambridge, UK), anti-E7 (1:500, ab308296; Abcam), anti-p53 (1:1000, 10442-1-AP; Proteintech), anti-p21 (1:1000, 10355-1-AP; Proteintech) and anti-E6AP/UBE3A (1:1000, 10344-1-AP; Proteintech). The membranes were then incubated with the appropriate secondary antibodies: horseradish peroxidase-conjugated goat anti-mouse IgG (1:5000, SA00001-1; Proteintech) or goat anti-rabbit IgG (1:5000, SA00001-2; Proteintech). The signals were visualised via a high-signal ECL western blotting substrate (180–5001; Tanon, Beijing, China) and detected using a Tanon 5200 imaging system (Tanon).

**Fluorescence microscopy analysis**

HEK293T cells were co-transfected with plasmids encoding d2EGFP, EGFP Fc-Nanobody and various Myc-tagged Trim21-based constructs. The fluorescence was detected 48 h later via a BX51 microscope (Olympus, Japan). The intensity of EGFP in at least five fields was quantified using ImageJ, and this experiment was repeated three times.

**Luciferase reporter assay**

CaSki cells were co-transfected with pGL3-p21-luc, pRL-TK and plasmids encoding E6 Fc-Nanobody and TRIM21 (ΔBB) for 48 h. The cells were then harvested, lysed with 5× passive buffer and subjected to a dual-luciferase reporter assay according to the manufacturer’s instructions (Promega, Madison, USA).

**Cell Counting Kit-8 assay**

CaSki cells co-transfected with plasmids encoding E6 Fc-Nanobody and TRIM21 (ΔBB) were seeded in 96-well plates at 5000 cells/well. These cells were then incubated with Cell Counting Kit-8 (CCK-8) solution (Beyotime Biotechnology) for 2 h at different time points (0, 24, 48 or 72 h). The 0 h time point was 6 h after the cells were seeded in the plates. The product was then quantified by spectrophotometry at a wavelength of 450 nm using a microplate reader (Bio-Rad, Hercules, USA). These experiments were performed with six replicates and were repeated three times.

**Colony formation assay**

CaSki cells cotransfected with plasmids encoding E6 Fc-Nanobody and TRIM21 (ΔBB) were seeded in 6-well plates (1000 cells/well). After 6 days of incubation at 37°C, the cells were fixed with 4% paraformaldehyde (Beyotime Biotechnology) for 10 min at room temperature, followed by staining with 0.2% crystal violet (Beyotime Biotechnology) for 10 min at room temperature. Images were captured using an iPhone 11 camera (Apple, Inc., Cupertino, USA), and the number of colonies (≥50 cells) was manually counted using a light field microscope (CKX53; Olympus, Tokyo, Japan).

**Reference**

1 Li C, Lu W, Yang L, Li Z, Zhou X, Guo R, Wang J*, et al.* MKRN3 regulates the epigenetic switch of mammalian puberty via ubiquitination of MBD3. Natl Sci Rev 2020, 7: 671-685
